# Supplementary material for: Tough double network hydrogels with rapid self-reinforcement and low hysteresis based on highly entangled networks
Source: Nat Commun. 2024 Feb 13;15:1344. doi: 10.1038/s41467-024-45485-8 (PMC10864390; doi:10.1038/s41467-024-45485-8)
Supplement: Supplementary file 1 — Supplementary Information [file 41467_2024_45485_MOESM1_ESM.pdf]

Supplementary Information

Tough double network hydrogel with rapid self-reinforcement and low hysteresis based on highly entangled networks

Ruixin Zhu,[1,2] Dandan Zhu,[1,2] Zhen Zheng,[1,2] Xinling Wang,[*,1,2]

[1] School of Chemistry and Chemical Engineering, Shanghai Jiao Tong University, Shanghai 200240, China

[2] State Key Laboratory of Metal Matrix Composites, Shanghai Jiao Tong University, Shanghai 200240, China

*Corresponding authors. E-mail address: xlwang@sjtu.edu.cn (X. Wang)

Contact information for authors.

Ruixin Zhu zhuruixin@sjtu.edu.cn

Dandan Zhu zhudan@sjtu.edu.cn

Zhen Zheng zzheng@sjtu.edu.cn

The file includes:

Supplementary Tables 1 to 3

Supplementary Figures 1 to 21

Supplementary Tables

Supplementary Table 1 Formulation of high entanglement first network hydrogels (HEDN1st)

Hydrogel	AAm [g]	AMPS [g]	MBAA [mg]	I2959 [mg]	H_2O [mL]
HEDN1st-0.2	8.63	0.2	0.189	0.110	4.41
HEDN1st-0.4	8.63	0.4	0.190	0.111	4.44
HEDN1st-0.6	8.63	0.6	0.191	0.111	4.47
HEDN1st-0.7	8.63	0.7	0.192	0.112	4.49
HEDN1st-0.8	8.63	0.8	0.193	0.112	4.51
HEDN1st-1.0	8.63	1.0	0.195	0.113	4.54
HEDN1st-1.5	8.63	1.5	0.198	0.115	4.63
HEDN1st-2.0	8.63	2.0	0.202	0.117	4.72
HEDN1st-3.0	8.63	3.0	0.210	0.122	4.89
HEDN1st-4.0	8.63	4.0	0.217	0.126	5.07

Supplementary Table 2 Preparation formula of HEDN1st-0.8 hydrogels with different entanglement degree. W is the molar ratio of water to total monomer

Hydrogel	AAm [g]	AMPS [g]	MBAA [mg]	I2959 [mg]	H_2O [mL]
HEDN1st-0.8-W2	8.63	0.8	0.193	0.112	4.51
HEDN1st-0.8-W4	8.63	0.8	0.193	0.112	9.02
HEDN1st-0.8-W6	8.63	0.8	0.193	0.112	13.53
HEDN1st-0.8-W8	8.63	0.8	0.193	0.112	18.04
HEDN1st-0.8-W10	8.63	0.8	0.193	0.112	22.55

Supplementary Table 3 Basic parameters of hydrogels with strain-stiffening characteristics

	References	Water content (%)	Stress at break (MPa)	Strain-stiffening capability
HEDN-0.8	This work	89	3.0	47.5
HEDN-AAc-3.0	This work	92	2.1	26.7
P(AM-AN-MA)/Fe^{3+}	Adv. Funct. Mater. 2023, 2210224.	84	1.4	28
PVAm/EGDE-Zn^{2+}	ACS Appl. Mater. Interfaces 2022, 14, 27, 31354–31362.	85	1.3	10
CNF-g-pHEMA/pHEMA	Cellulose volume 28, pages1489–1497 (2021).	67.9	2.1	15.4
QCS-AMP/PAAm	J. Mater. Chem. A, 2021, 9, 1835-1844.	75	0.35	12.3
SRC-7-20	Adv. Funct. Mater. 2021, 31, 2104139.	60	5	32
PAAS/PAAm	ACS Appl. Mater. Interfaces 2022, 14, 47148−47156.	75	0.8	13

Supplementary Figures

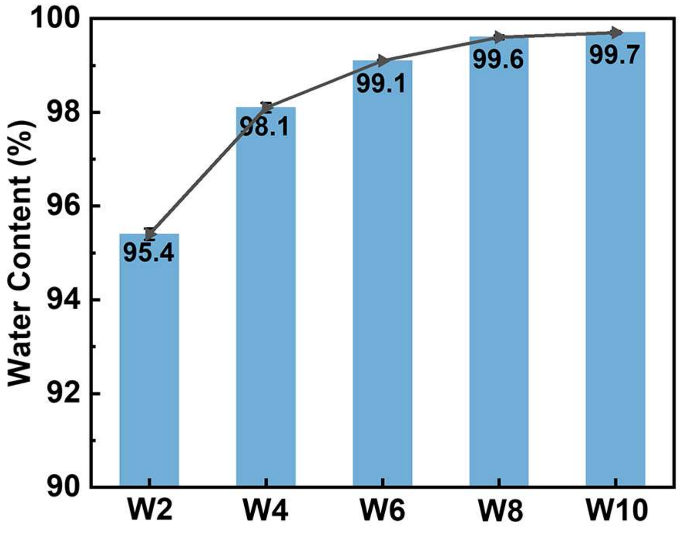

Supplementary Figure 1 Water content of fully swollen HEDN1st-0.8-W hydrogels with different W. W is the molar ratio of water to total monomer. Error bars represent mean +/- standard deviation (n=5).

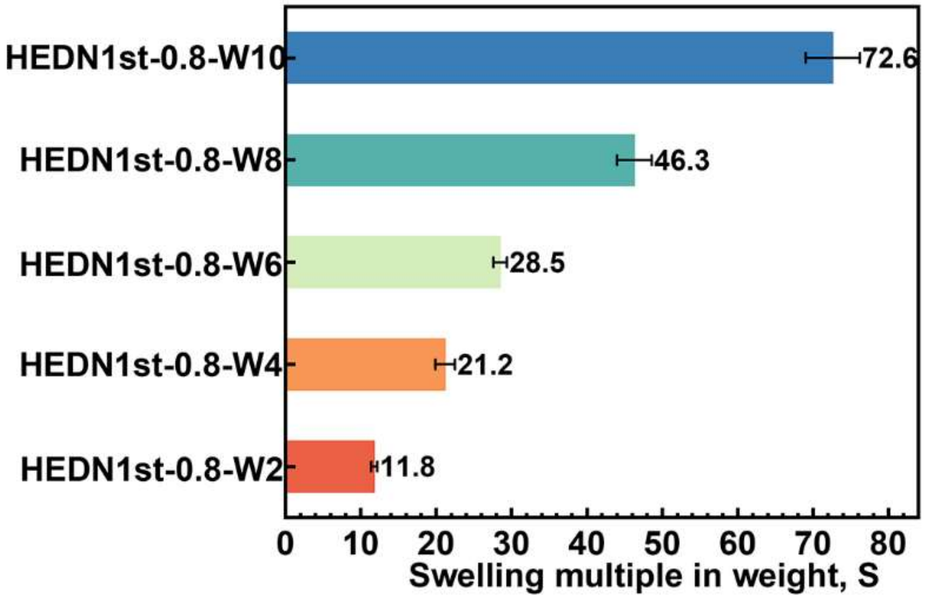

Supplementary Figure 2 Swelling multiple in weight of HEDN1st hydrogels obtained at different concentrations in 4 M AAm solution. W is the molar ratio of water to total monomer, e.g., W2 means that the molar ratio of water to total monomer is 2. Error bars represent mean +/- standard deviation (n=5).

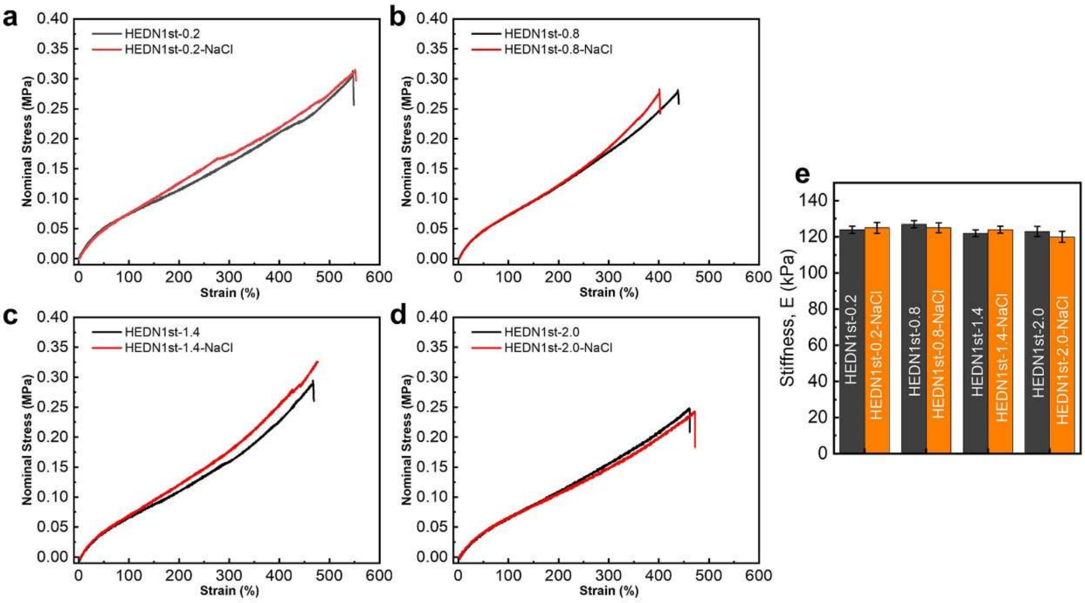

Supplementary Figure 3 Tensile stress-strain curves of HEDN1st-0.2 (**a**), HEDN1st-0.8 (**b**), HEDN1st-1.4 (**c**) and HEDN1st-2.0 (**d**) hydrogels swollen by water or salt solutions (3 M NaCl). The stiffness of these hydrogels (**e**). Polymer fraction is 11%. Error bars represent mean +/- standard deviation (n=5).

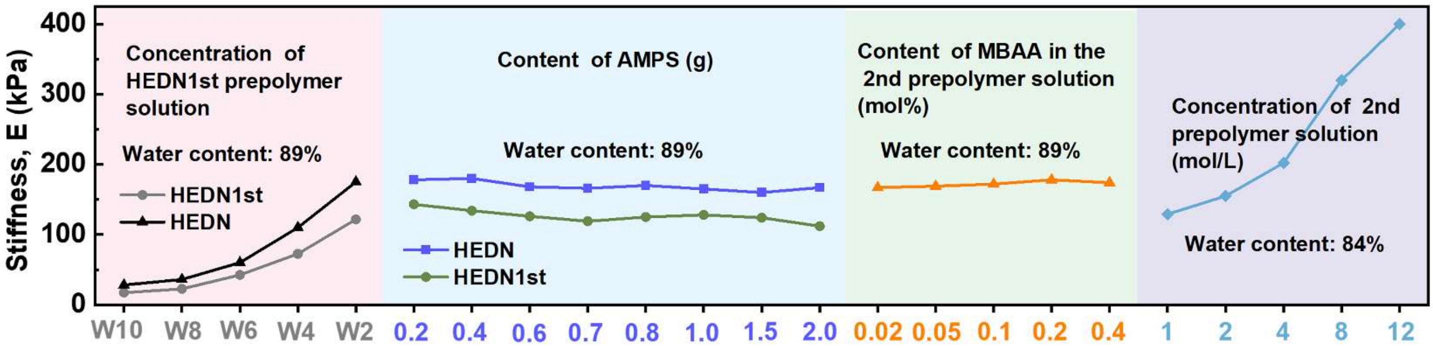

Supplementary Figure 4 Effects of prepolymer concentration, crosslinker dosage and AMPS dosage on stiffness of HEND1st and HEDN hydrogel, respectively.

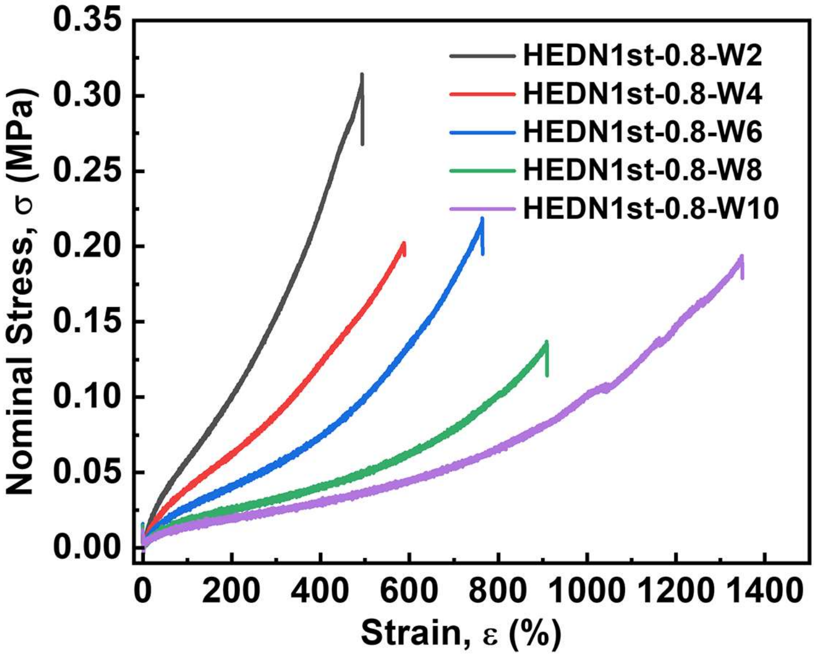

Supplementary Figure 5 Tensile stress-strain curves of HEDN1st-0.8 hydrogels with different degree of entanglements. Water content: 89%.

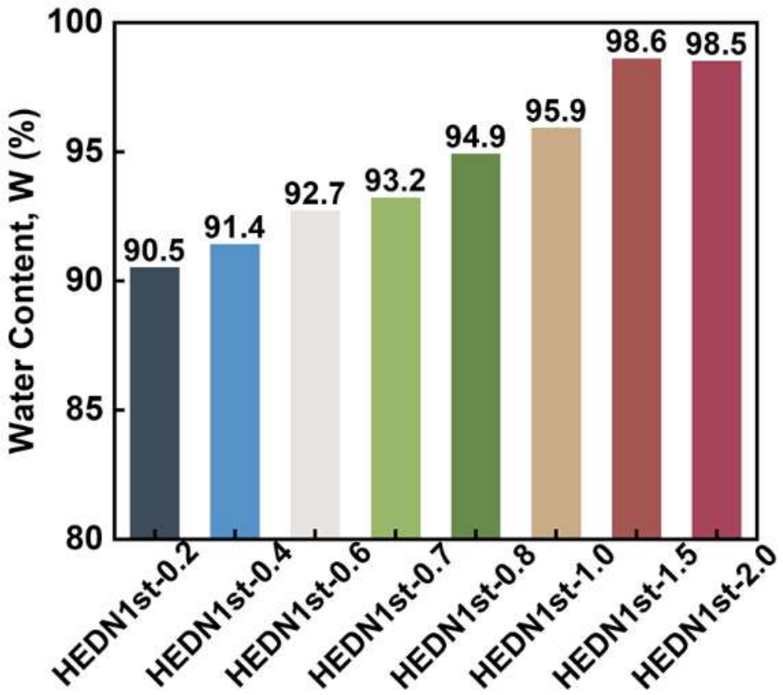

Supplementary Figure 6 Water content of HEDN1st hydrogels with different AMPS contents.

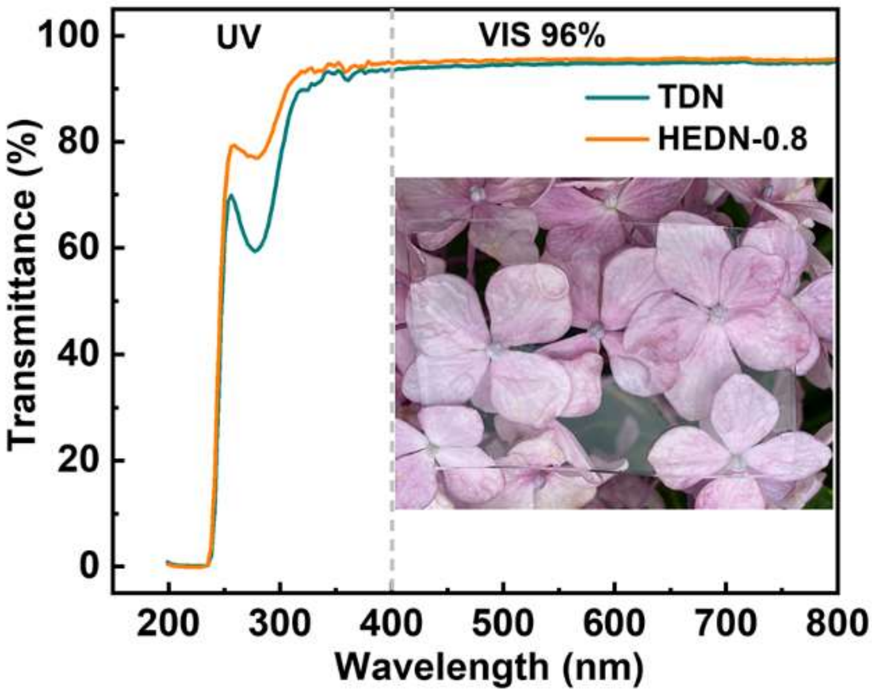

Supplementary Figure 7 UV- visible light transmission spectra of TDN and HEDN-0.8 hydrogels. Inner image: Photo of HEDN-0.8 hydrogel on flowers.

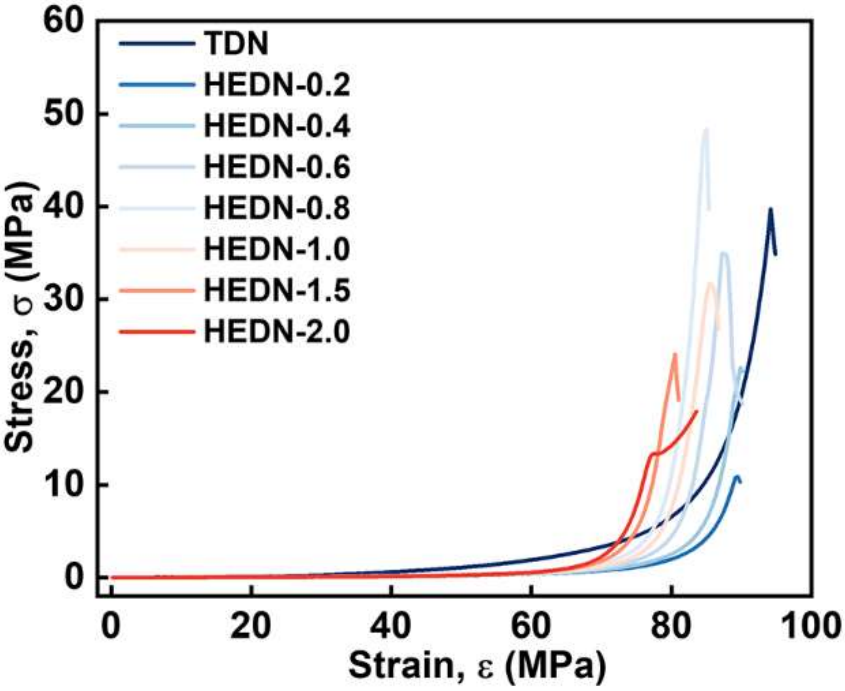

Supplementary Figure 8 Compressive stress-strain curves of TDN hydrogel and HEDN hydrogels with different AMPS contents.

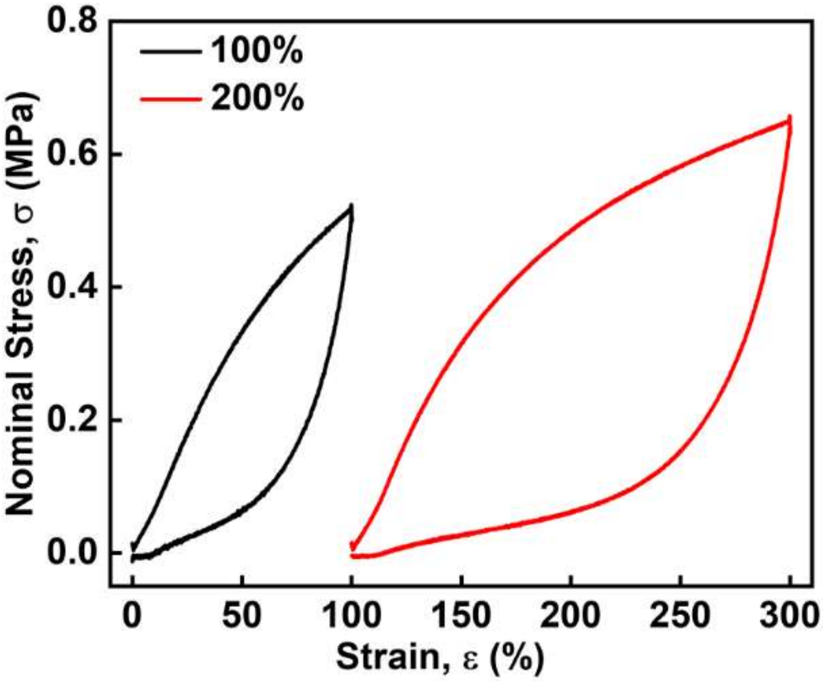

Supplementary Figure 9 Single cyclic tensile curves of TDN hydrogel at 100% and 200% strain.

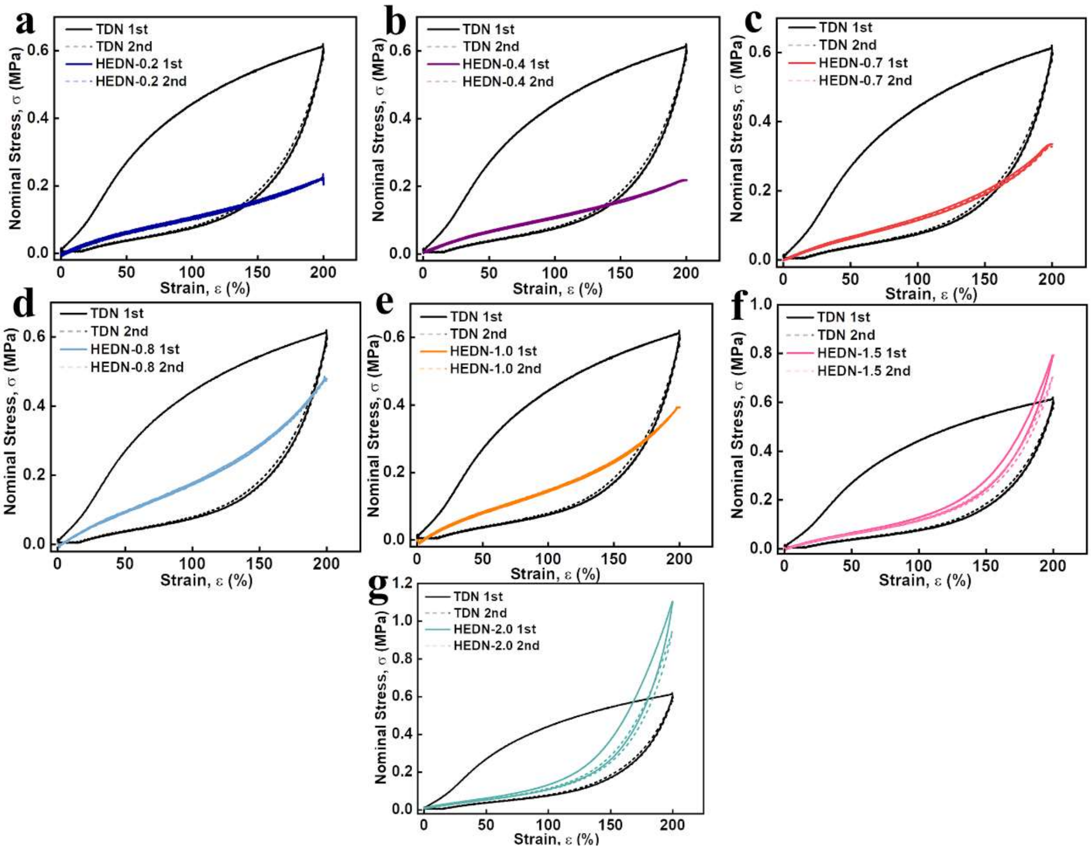

Supplementary Figure 10 Comparison of the two-cycle tensile curves of HEDN

hydrogels with different AMPS contents and TDN hydrogel at 200% strain.

The reversibility of HEDN-1.5 and HEDN-2.0 hydrogel are 90% and 82%, respectively. Due to the low fracture strain of the two hydrogels, which had partially broken polymer chains when stretched to 200%.

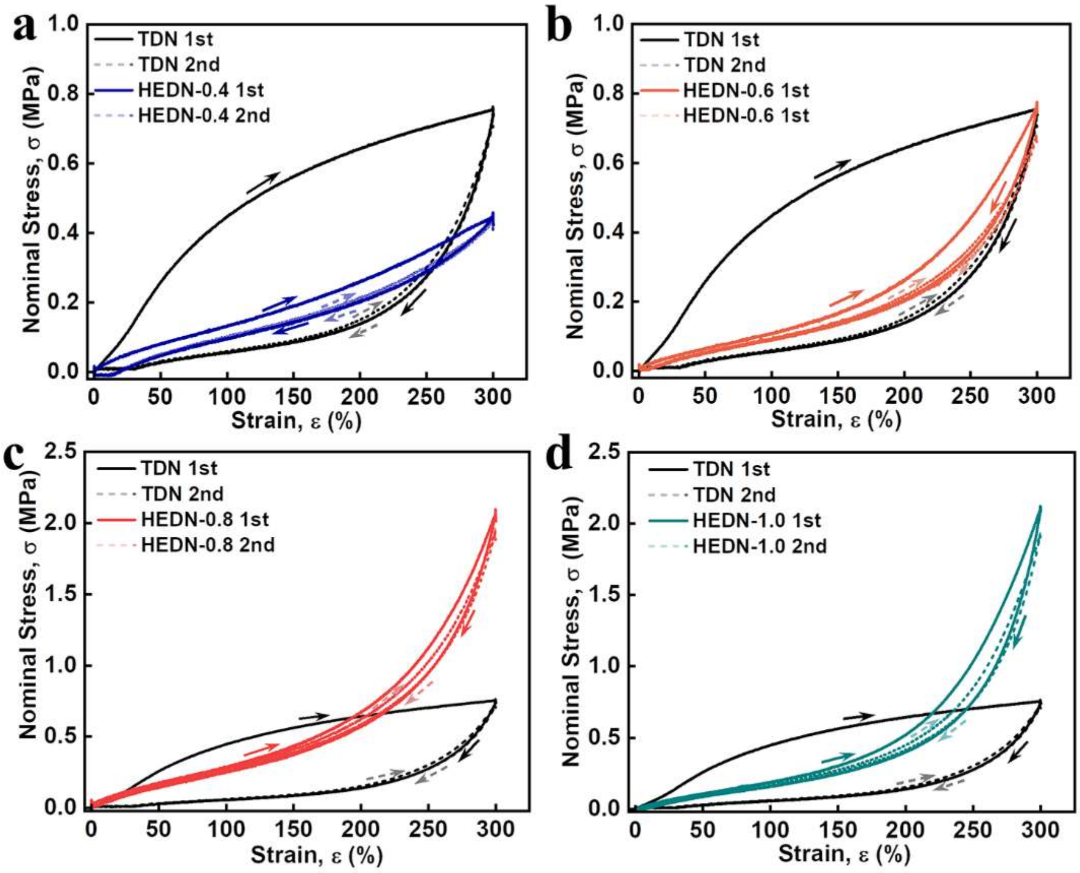

Supplementary Figure 11 Comparison of the two-cycle tensile curves of HEDN hydrogels with different AMPS contents and TDN hydrogel at 300% strain.

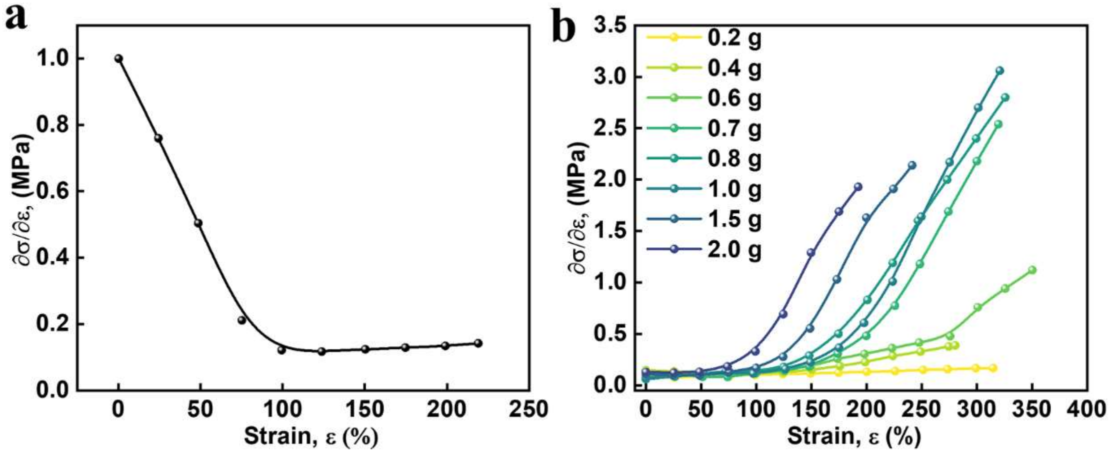

Supplementary Figure 12 Differential modulus (∂σ/∂λ) of TDN hydrogel (a) and HEDN hydrogels (b) with different AMPS contents against strain ε.

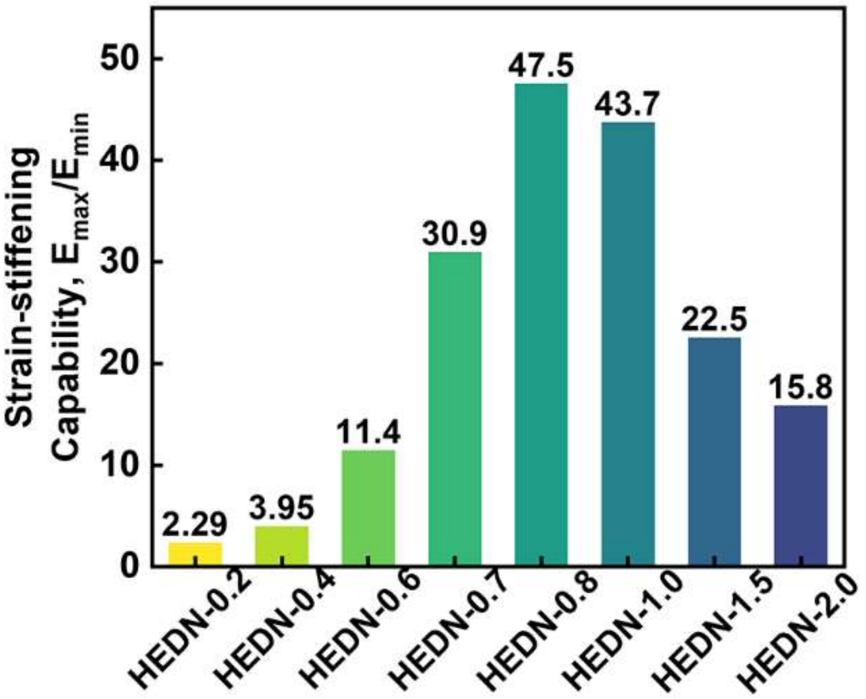

Supplementary Figure 13 Strain-stiffening capability of HEDN hydrogels with different AMPS contents.

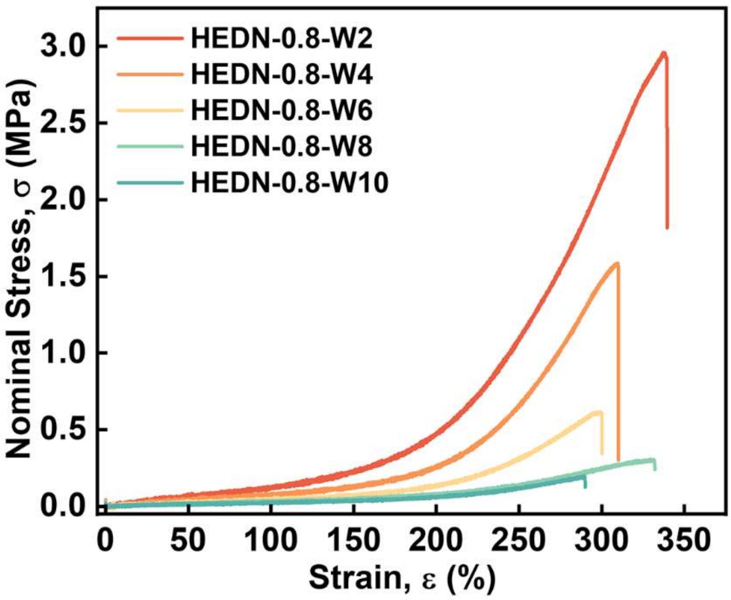

Supplementary Figure 14 Tensile stress-strain curves of HEDN-0.8 hydrogels with different degree of entanglements.

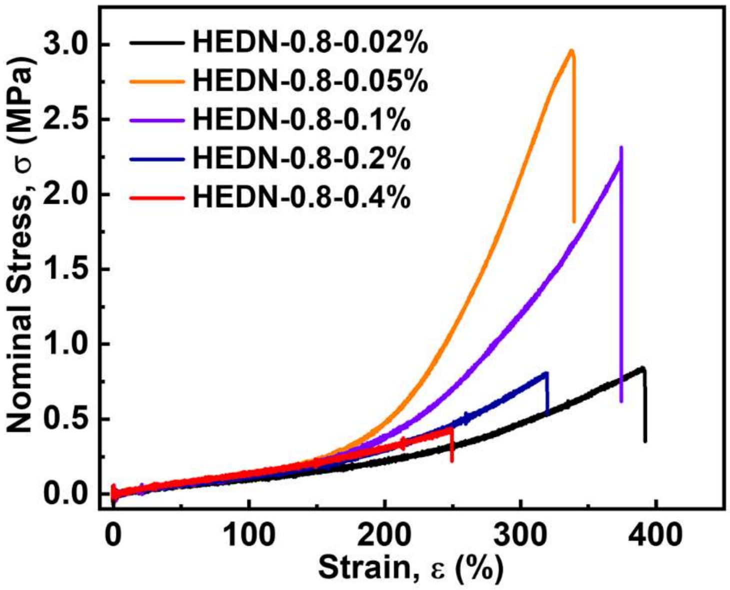

Supplementary Figure 15 Tensile stress–strain curves of HEDN-0.8 hydrogels with various contents of 2nd network MBAA. Water content: 89%.

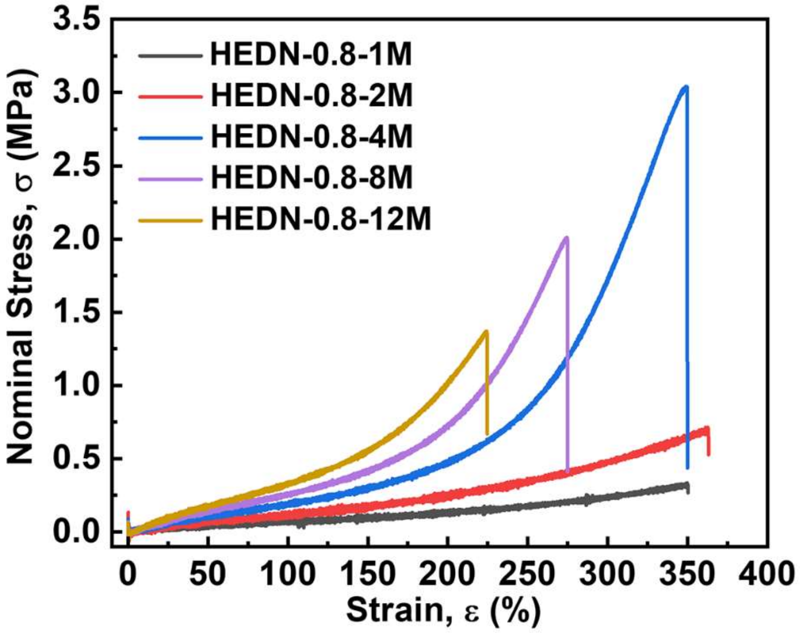

Supplementary Figure 16 Tensile stress–strain curves of HEDN-0.8 hydrogels with various concentrations of 2nd network monomer. Water content: 84%.

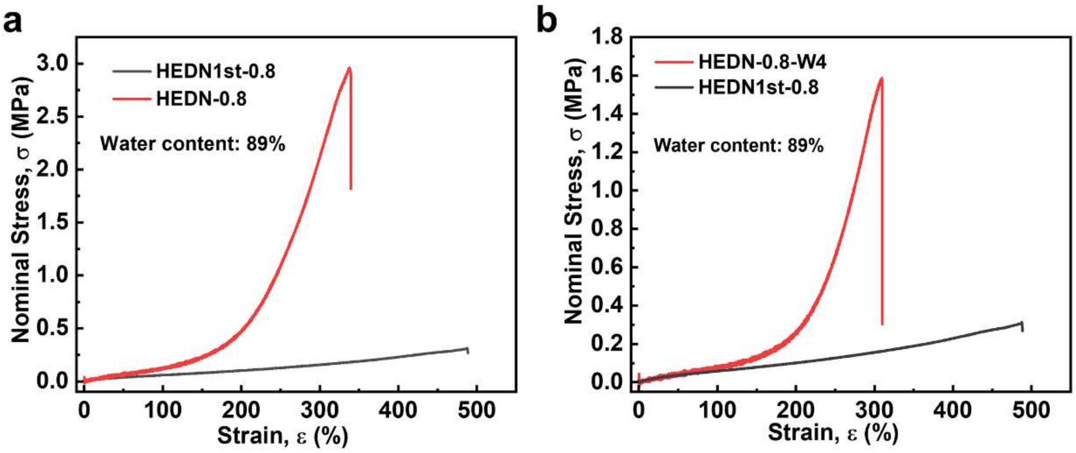

Supplementary Figure 17 a Tensile stress–strain curves of HEDN1st-0.8 hydrogel and HEDN-0.8 hydrogels with the same level of water content. **b** Tensile stress–strain curves of HEDN1st-0.8 hydrogel and HEDN-0.8-W4 hydrogels with the same level of water content and stiffness.

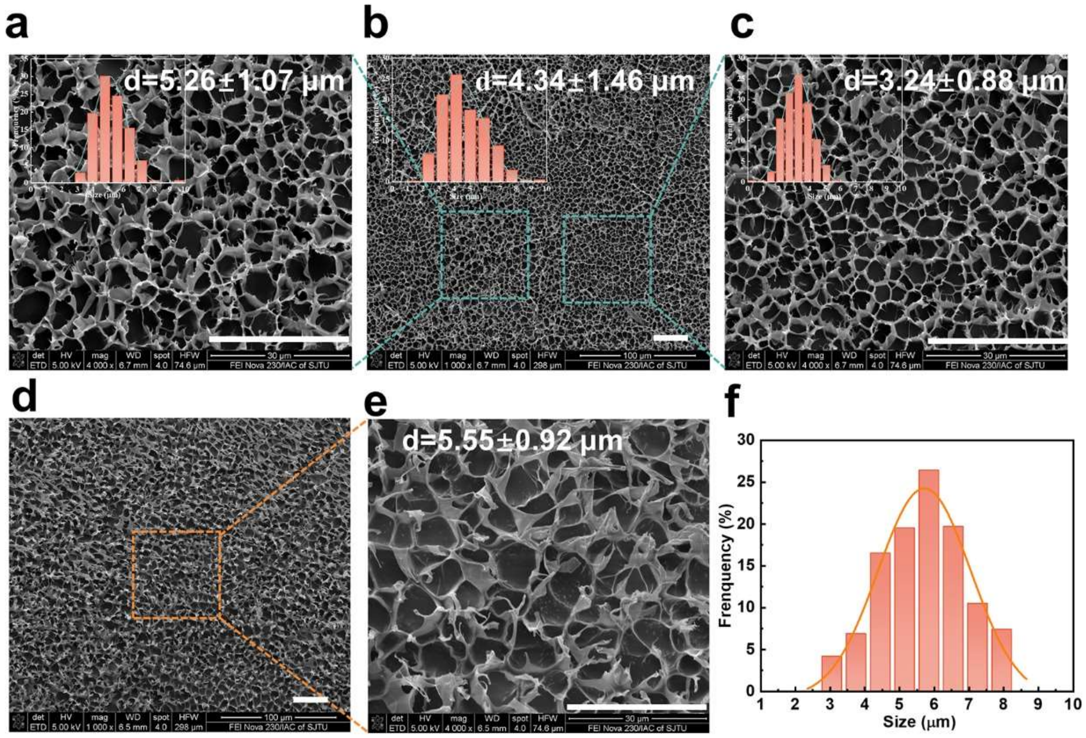

Supplementary Figure 18 a SEM images of large-pore microdomains in TDN hydrogel. inner image is a pore size statistical distribution. **b** SEM images of large and small pore microdomains in TDN hydrogels. **c** SEM images of small-pore microdomains in TDN hydrogel. **d,e** SEM images of HEDN-0.8 hydrogels at different magnifications. **f** Pore size statistical distribution of HEDN-0.8. Scale bars, 30 μm.

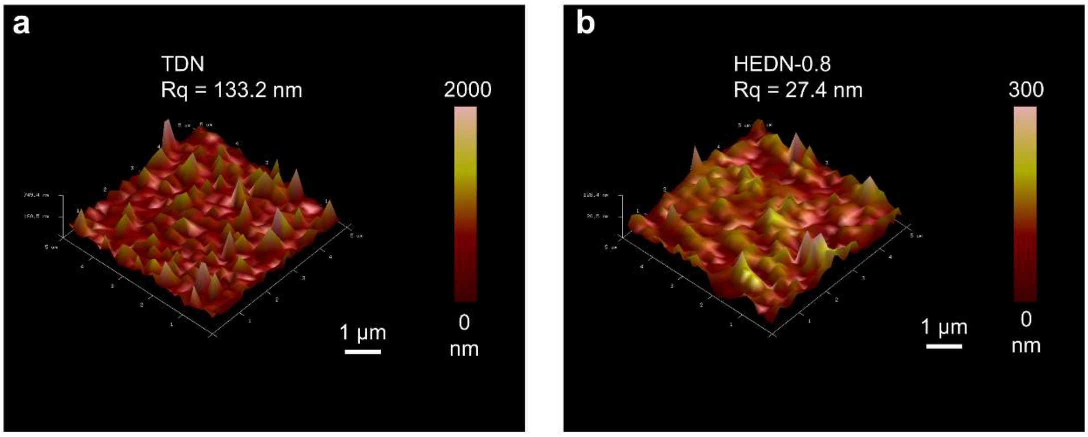

Supplementary Figure 19 AFM height images of TDN hydrogel and HEDN-0.8 hydrogel in wet state.

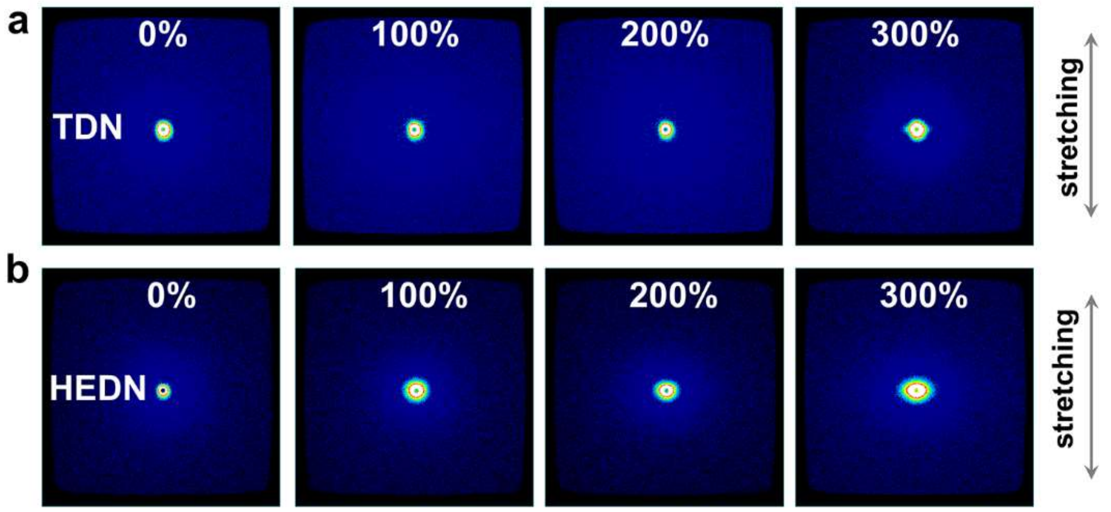

Supplementary Figure 20 SAXS patterns of TDN (a) and HEDN-0.8 hydrogels (b) with different strains during in situ stretching. The arrow indicates the stretching direction.

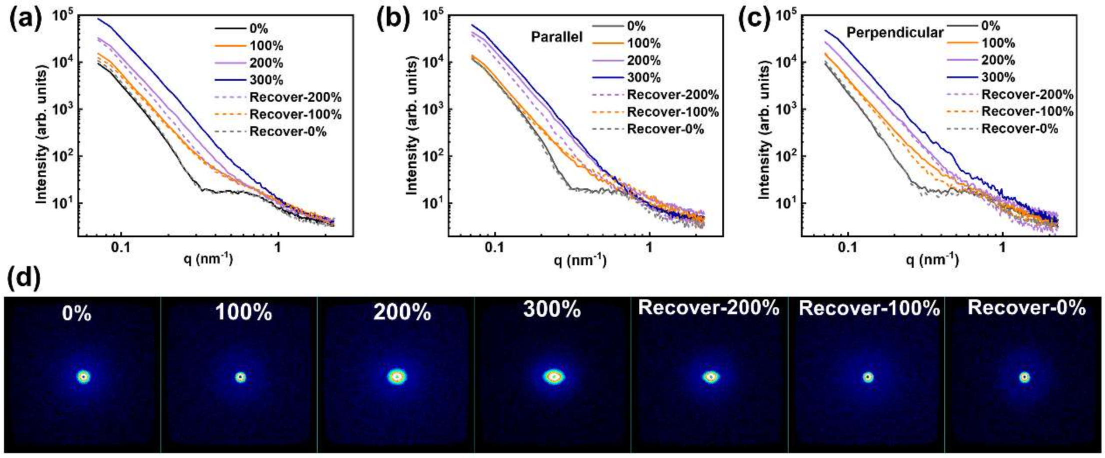

Supplementary Figure 21 a One-dimensional SAXS profiles of HEDN-0.8 hydrogel. **b-c** 1D SAXS profiles of HEDN-0.8 hydrogels with different strains in parallel (b) and perpendicular (c) directions. **d** 2D SAXS patterns of HEDN-0.8 hydrogels with different strains.
