# Supplementary material for: Tough double network hydrogels with rapid self-reinforcement and low hysteresis based on highly entangled networks
Source: Nat Commun. 2024 Feb 13;15:1344. doi: 10.1038/s41467-024-45485-8 (PMC10864390; doi:10.1038/s41467-024-45485-8)
Supplement: Supplementary file 3 — Description of Additional Supplementary Files [file 41467_2024_45485_MOESM3_ESM.pdf]

Description of Additional Supplementary Files

File Name: Supplementary Movie 1
Description: Interference color change of TDN hydrogel under polarized mode in-situ stretching. The interference color gradually appeared and brightened with the increase of tensile strain.

File Name: Supplementary Movie 2
Description: Interference color change of HEDN-0.8 hydrogel under polarized mode in-situ stretching. The interference color gradually appeared with the increase of tensile strain, and then gradually brightened, and finally showed a color change in accordance with Michel-Levy chart.

File Name: Supplementary Movie 3
Description: Crack growth of notched TDN hydrogel.

File Name: Supplementary Movie 4
Description: Crack blunting and growth of notched HEDN-0.8 hydrogel.

File Name: Supplementary Movie 5
Description: Interference color distribution of notched TDN hydrogel under polarized mode in-situ stretching. The Interference color is distributed only around the notch.

File Name: Supplementary Movie 6
Description: Interference color distribution of notched HEDN-0.8 hydrogel under polarized mode in-situ stretching. The interference color is distributed in the bulk of the gel, and the brightness of the interference color is almost the same as that around the notch.
